# Chemical Ecology of Cave-Dwelling Millipedes: Defensive Secretions of the Typhloiulini (Diplopoda, Julida, Julidae)

**DOI:** 10.1007/s10886-017-0832-1

**Published:** 2017-03-16

**Authors:** Slobodan E. Makarov, Michaela Bodner, Doris Reineke, Ljubodrag V. Vujisić, Marina M. Todosijević, Dragan Ž. Antić, Boyan Vagalinski, Luka R. Lučić, Bojan M. Mitić, Plamen Mitov, Boban D. Anđelković, Sofija Pavković Lucić, Vlatka Vajs, Vladimir T. Tomić, Günther Raspotnig

**Affiliations:** 1 Institute of Zoology, University of Belgrade - Faculty of Biology, Studentski Trg 16, Belgrade, 11000 Serbia; 2 Institute of Zoology, University of Graz, Universitätsplatz 2, 8010 Graz, Austria; 3 Faculty of Chemistry, University of Belgrade, Studentski trg 12-16, Belgrade, 11000 Serbia; 4 Institute of Biodiversity and Ecosystem Research, Department of Animal Diversity and Resources, Bulgarian Academy of Sciences, 2 Gagarin Street, 1113 Sofia, Bulgaria; 5 Department of Zoology and Anthropology, Sofia University “St. Kliment Ohridsky”, Sofia, Bulgaria; 6 Institute of Chemistry, Technology and Metallurgy, University of Belgrade, Studentski trg 12-16, Belgrade, 11000 Serbia; 7 Research Unit of Osteology and Analytical Mass Spectrometry, Medical University, Children’s Hospital, Auenbruggerplatz 30, 8036 Graz, Austria

**Keywords:** Typhloiulini, *Typhloiulus*, *Serboiulus*, *Lamellotyphlus*, 2-ethyl-1,4-benzoquinone, quinone millipedes, chemical defense, chemosystematics

## Abstract

Cave animals live under highly constant ecological conditions and in permanent darkness, and many evolutionary adaptations of cave-dwellers have been triggered by their specific environment. A similar “cave effect” leading to pronounced chemical interactions under such conditions may be assumed, but the chemoecology of troglobionts is mostly unknown. We investigated the defensive chemistry of a largely cave-dwelling julid group, the controversial tribe “Typhloiulini”, and we included some cave-dwelling and some endogean representatives. While chemical defense in juliform diplopods is known to be highly uniform, and mainly based on methyl- and methoxy-substituted benzoquinones, the defensive secretions of typhloiulines contained ethyl-benzoquinones and related compounds. Interestingly, ethyl-benzoquinones were found in some, but not all cave-dwelling typhloiulines, and some non-cave dwellers also contained these compounds. On the other hand, ethyl-benzoquinones were not detected in troglobiont nor in endogean typhloiuline outgroups. In order to explain the taxonomic pattern of ethyl-benzoquinone occurrence, and to unravel whether a cave-effect triggered ethyl-benzoquinone evolution, we classed the “Typhloiulini” investigated here within a phylogenetic framework of julid taxa, and traced the evolutionary history of ethyl-benzoquinones in typhloiulines in relation to cave-dwelling. The results indicated a cave-independent evolution of ethyl-substituted benzoquinones, indicating the absence of a “cave effect” on the secretions of troglobiont Typhloiulini. Ethyl-benzoquinones probably evolved early in an epi- or endogean ancestor of a clade including several, but not all *Typhloiulus* (basically comprising a taxonomic entity known as “*Typhloiulus* sensu stricto”) and *Serboiulus*. Ethyl-benzoquinones are proposed as novel and valuable chemical characters for julid systematics.

**Electronic supplementary material:**

The online version of this article (doi:10.1007/s10886-017-0832-1) contains supplementary material, which is available to authorized users.

## Introduction

There is a need for a better understanding of the functional-ecological architecture of cave organisms (Romero 2009). In many troglobionts, character differentiation is related to the life in caves, and the morpho-anatomy of cave-dwellers is frequently characterized by regression or modification in light-sensitive structures such as eyes, loss of pigmentation, and enlargement or elongation of body appendices. Many of these modifications obviously arise late in the ontogenetic development (Romero 2009). In evolutionary terms, cave environments have clearly and rapidly affected the morphology of troglobionts. Interestingly, this “cave effect” leads to a stunningly homogenous phenology in different arthropod taxa, sometimes even masking phylogenetic relations (Cruz-López et al. 2016). Many of these arthropods are chemically still well-defended, and chemical defense appears to persist when species become cavernicolous. The defensive chemistry of troglobionts, however, has only been elucidated for a few species, mainly for some polydesmid millipedes, one example of cave-harvestmen and two carabids (Makarov et al. 2012; Shear et al. 2010a, 2010b; Vesović et al. 2015). A possible cave-effect on chemical defense, in terms of modification of defensive chemistry compared to non-cave dwellers of the same taxonomic group, has never been investigated in detail. In millipedes, troglobiism is frequent and various millipede groups contain cavernicolous taxa. Morphological adaptations of troglobiont diplopods are conspicuous, including a longer body, lighter body color, elongation of femora and tarsi, and these adaptations evolved independently in relatively unrelated millipede groups (Liu et al. 2017). One model group of millipede cave-dwellers is certainly the “Typhloiulini”, a putatively paraphyletic assemblage within leptoiulines, including both many cave-dwelling but also epi−/endogean species (Vagalinski et al. 2015).

Chemical defense in diplopods is generally considered a major survival strategy, and defensive glands in typhloiulines, as well as in typhloiuline troglobites, appear to be well-developed. In cave habitats, however, the predatory pressure may be considered lower compared to epigean ones. Cavernicolous typhloiulines apparently lack specialized predators in caves but still may be preyed on by other troglobites, such as spiders and coleopterans.

In the order Julida, no studies on the defensive chemistry of troglobites were available. For Polydesmida and Callipodida, initial investigations on cavernicolous species have recently been published, basically showing no influence of cave-living on the composition of secretions (Shear et al. 2007, 2010b). We here focus on the chemical defense of cavernicolous julids for the first time, comparing the secretions of both cavernicolous and non-cavernicolous “Typhloiulini”.

## Methods and Materials

### Collection of Species

**Fig. 1:**
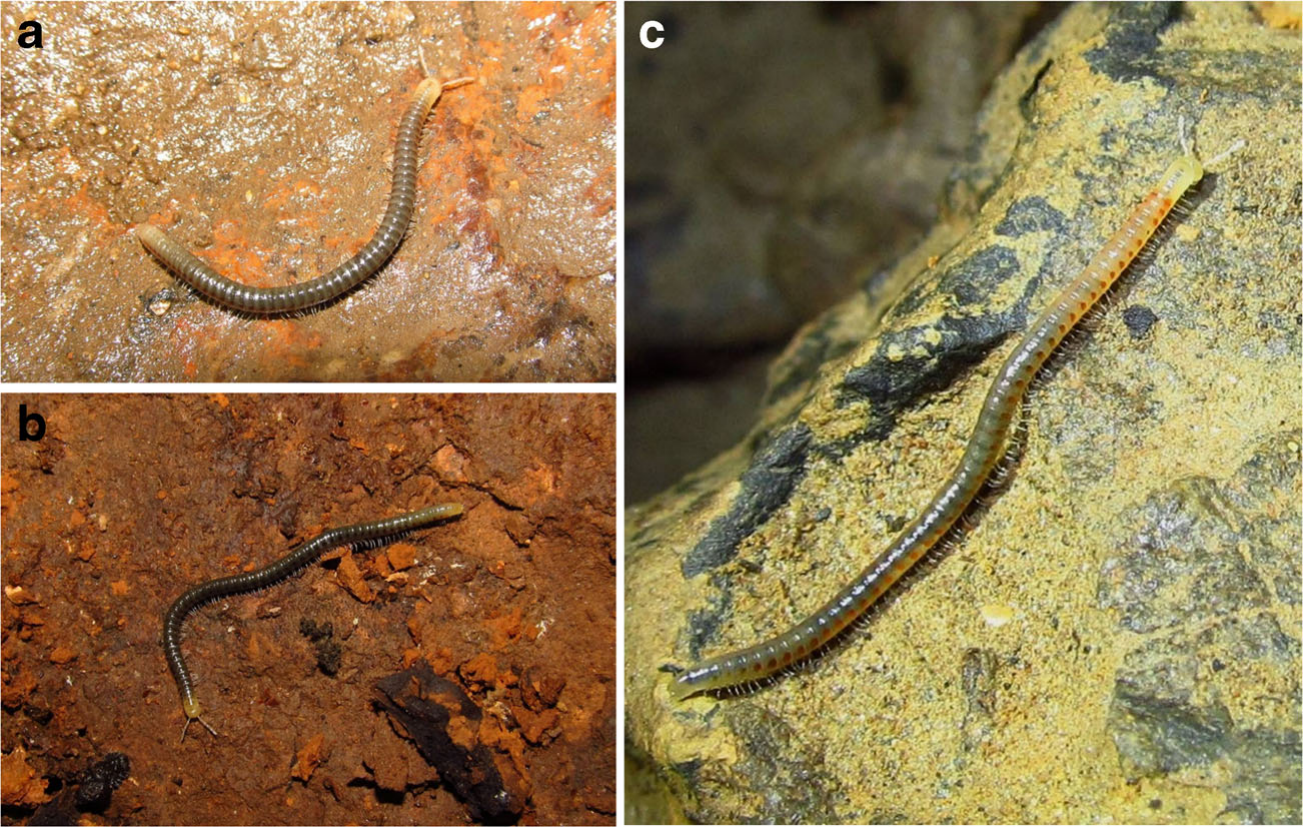
**a** *Typhloiulus serborum* Ćurčić et al. 2005, Devojačka Pećina Cave, vill. Podgorac, near Boljevac, East Serbia (photo D. Antić). **b** *Serboiulus deelemani* Strasser 1971, Vetrena Dupka Cave, v. Vlasi, Pirot, South Serbia (photo D. Antić). **c** *Lamellotyphlus sotirovi* Makarov et al. 2002, Buronov Ponor Cave, v. Golubinje, Miroč Mt., East Serbia (photo D. Antić). Defensive glands are visible

Adult individuals of 12 species representing 3 genera of typhloiulines were collected during four years (2012–2015) in East Serbia, Dalmatia (Croatia) and Bulgaria (Table 1). We included an undescribed species, “Typhloiulini” sp. n., and this preliminary designation will be used throughout the text. Individuals of three of the species, *Typhloiulus serborum*, *Serboiulus deelemani*, and *Lamellotyphlus sotirovi,* are pictured in Fig. 1.

**Table 1:** Details of species collected

Species	Locality and collector	Date of collection	No. specimens	Ecology
*Lamellotyphlus sotirovi*	Buronov Ponor Pit, Mt. Miroc, E Serbia; 44°33′31.04″N, 22°15′40.56″E; 290 m (D. Antić & Đ. Marković)	June 22, 2015	5 ♂, 5 ♀	troglobiont
*Serboiulus deelemani*	Vetrena Dupka Cave, Vlasi Village, near Pirot, S Serbia; 43° 0′ 11.20″N, 22° 37′ 55.70″E; 561 m (D. Antić)	July 2014	5 ♂, 5 ♀	troglobiont
*Serboiulus kresnik*	Gornja Lenovačka Pećina Cave, Lenovac Village, Mt. Tupižnica, E Serbia; 43° 46′ 30.71″N, 22° 9′ 34.15″E; 335 m (D. Antić & S. Ćurčić)	July 2014	5 ♂, 5 ♀	troglobiont
*Serboiulus lucifugus*	Prekonoška Pećina Cave, Prekonoga Village, near Svrljig, S Serbia; 43° 22′ 49.3″N, 22° 6′ 7.7″E; 699 m (D. Antić)	July 2014	5 ♂, 5 ♀	troglobiont
*Typhloiulus bureschi*	Western Stara planina Mts., Iskar Gorge, Lakatnik railway station, Svinskata dupka Cave (Sofia District, Bulgaria); 43° 05′ 17.03″N, 23° 22′ 20.94″E; 480 m (B. Vagalinski & P. Mitov)	April 4, 2013	4 ♀	troglobiont
*Typhloiulus georgievi*	Central Stara planina Mts., v. Golyama Zhelyazna, Toplya Cave (Lovech District, Bulgaria); 42° 56′ 53.88″N, 24° 29′ 15.00″E; 466 m (B. Vagalinsk & S. Lukanov)	Nov. 11, 2014	4 ♂, 4 ♀	troglobiont
*Typhloiulus lobifer*	Minjera Cave, near Škrip, Brač, Croatia; 43° 21′ 41.31″N, 16° 36′ 22.39″E; 203 m (T. Radja & D. Antić)	Sept. 2014	4 ♂, 3 ♀	troglobiont
*Typhloiulus* aff. *lobifer*	Jama na Boroviku Pit, Hvar, Croatia; 91 43° 8.8′ 22″N, 16° 41′ 14.64″E (T. Radja)	Nov. 09, 2013	2 ♂, 3 ♀	troglobiont
*Typhloiulus nevoi*	Petrlaška Pećina Cave, Petrlaš Village, Dimitrovgrad, E Serbia; 43° 4′ 28.22″N, 22° 47′ 46.85″E; 697 m (D. Antić)	June 2014	3 ♂, 3 ♀	troglobiont
*Typhloiulus orpheus*	Western Rhodopi Mts., v. Trigrad, near Dyavolskoto garlo Cave (Smolyan District, Bulgaria) 41°36′54.51″N, 24°22′48.94″E; 1250–1300 m (B. Vagalinski)	May 27, 2014	1 ♂, 2 ♀	epi−/endogean
*Typhloiulus serborum*	Samar Cave System, Kopajkošara Village, near Niš, SE Serbia; 43° 26′ 45.40″N, 21° 58′ 34.50″E; 500 m (D. Antić)	July 2014	2 ♂	endogean + troglobiont
*Typhloiulini* sp. n.	between Belitsa and Borovo (Plovdiv District, Laki Municipality, Bulgaria); 41°50′20.94″N, 24°51′35.74″E; 695 m (B. Vagalinski & P. Mitov)	May 1, 2015	2 ♂, 8 ♀	epi−/endogean

### Chemical Analyses

The defensive secretions of typhloiulines were extracted in methylene chloride or hexane (0.5 ml), respectively, for 3 min. To avoid the effects of any oxidation and degradation, a portion of each extract was analyzed by gas chromatography-mass spectrometry (GC-MS) immediately after preparation.

Secretions of *S. lucifugus, S. kresnik, S. deelemani, T. serborum, T. nevoi*, *T.* aff. *lobifer*, *T. lobifer* and *L. sotirovi* (Table 2) were analysed in the laboratory of the Faculty of Chemistry, University of Belgrade, Belgrade, Serbia. GC and GC-MS analyses were performed on an Agilent 7890A GC system (Agilent Technologies, Santa Clara, CA, USA) equipped with a 5975C inert XL EI/CI MSD and a FID detector connected by capillary flow technology 2-way splitter with make-up gas. An HP-5MSI capillary column (Agilent Technologies, 0.25 mm i.d., 30 m length, 0.25 μm film thickness) was used. Samples were injected in splitless mode and the injection volume was 1 μl. Carrier gas was helium at a flow rate was 1.6 ml/min at 40 °C in constant pressure mode. The column temperature was held at 40 °C for 1 min and then programmed at 10 °C/min to 300 °C and held for 8 min. Mass spectra were acquired in electron ionization mode (EI) with ion energy of 70 eV and chemical ionization (CI) mode with ion energy of 150 eV. CI mass spectra were obtained in positive mode with isobutane as the reagent gas. The scan range was *m/z* 35–550 in EI mode, and *m/z* 60–550 in CI mode.

**Table 2:** Gas chromatographic profiles of defensive secretions of typhloiuline species investigated; secretion profiles for species are given as % of peak area of compounds relative to the total area of secretion compounds and refer to characteristic profiles of a single individual or profiles from pooled (2–3) individuals

			Species^a^											
Peak no	RI	Compound	Tb	Tg	Tal	Tl	Tn	To	Ts	Tspn^b^	Sd	Sk	Sl	Ls
1	917	1,4-benzoquinone	0.9	1.9	-	-	-	-	-	0.1	-	-	-	-
2	977	phenol	-	-	-	-	-	-	-	0.7	-	-	-	-
3	1015	2-methyl-1,4-benzoquinone	35.2	25.7	0.6	trace	11.4	0,1	13.1	31.8	18.8	25.0	14.6	-
4	1071	4-methylphenol	-	-	-	-	-	-	0.1	31.8	0.4	-	-	-
5	1108	2-ethyl-1,4-benzoquinone	34.9	47.9	-	-	22.7	0,3	35.7	-	56.7	48.5	66.3	-
6	1120	2-hydroxy-3-methyl-1,4-benzoquinone	3.5	0.5	0.7	5.6	8.1	-	6.9	0.4	-	2.0	0.8	-
7	1177	4-ethylphenol	-	-	-	-	-	-	0.1	-	0.2	-	-	-
8	1182	2-methoxy-3-methyl-1,4-benzoquinone	21.1	20.2	81.9	74.4	6.7	-	13.2	8.1	14.4	7.0	1.4	-
9	1191	unidentified	0.1	-		-	-	-	-	-	-	-	-	-
10	1243	2-ethyl-3-methoxy-1,4-benzoquinone	1.3	2.3	0.5	-	3.0	-	1.8	-	2.4	0.8	0.1	-
11	1245	2-methoxy-1,4-benzoquinone	-	-	0.7	-	-	-	-	-	-	0.6	-	-
12	1317	unidentified	-	-	-	-	-	-	0.4	-	-	1.1	3.3	-
13	1319	2,3-dimethoxy-1,4-benzoquinone	2.7	1.4	6.0	3.2	15.9	-	2.1	trace	0.9	4.0	1.0	-
14	1341	2-methylhydroquinone	0.2	-	-	-	-	-	0.8	-	0.2	0.2	0.1	-
15	1341	2-methoxy-5-methyl-1,4-benzoquinone	-	-	0.3	-	-	-	-	0.7	-	0.1	-	2.0
16	1346	2-methoxy-6-methyl-1,4-benzoquinone	-	-	-	-	-	-	-	0.6	-	-	-	0.4
17	1349	unidentified	-	-	-	-	-	0,2	-	-	-	-	-	-
18	1375	2,3-dimethoxyhydroquinone	0.1	-	1.5	2.7	9.9	-	4.2	-	0.6	1.3	0.4	-
19	1386	2-methyl-3,4-methylenedioxyphenole	-	-	3.6	13.2	18.5	-	15.5	-	1.0	4.0	1.2	-
20	1411	2-hydroxy-3-methoxy-1,4-benzoquinone	-	-	-	-	-	-	-	0.2	-	-	-	-
21	1421	2,3-dimethoxy-5-methyl-1,4-benzoquinone	-	-	2.0	0.3	3.8	-	-	1.3	0.2	1.3	10.7	86.7
22	1422	unidentified	-	-	-	-	-	99,4	-	-	-	-	-	-
23	1436	2-ethyl-hydroquinone	-	-	-	-	-	-	4.0	-	2.7	0.9	-	-
24	1449	dimethoxy-methylhydroquinone /isomer 1	-	-	0.8	0.5	-	-	-	-	0.8	0.6	0.1	0.5
25	1455	methylparaben	-	-	1.1	-	-	-		0.8	-	-	-	-
26	1465	2,3,5,6-tetramethylhydroquinone	-	-	-	-	-	-	1.8	-	-	-	-	-
27	1499	dimethoxy-hydroxy-benzoquinone isomer	-	-	-	-	-	-	-	0.2	-	-	-	-
28	1511	2,6-dimethoxy-3-methyl-1,4-benzoquinone	-	-	-	-	-	-	-	-	-	-	-	10.0
29	1518	2-hydroxy-3-methoxy-5-methyl-1,4-benzoquinone	-	-	-	-	-	-	-	5.4	-	-	-	-
30	1522	2,3-dimethoxy-5,6-dimethyl-1,4-benzoquinone	-	-	-	-	-	-	-	-	-	1.6	-	-
31	1532	unidentified	-	-	-	-	-	-	-	0.6	-	-	-	-
32	1535	2,3-dimethoxy-5,6-dimethylhydroquinone	-	-	-	-	-	-	-	-	0.6	1.0	-	-
33	1598	unidentified	-	-	-	-	-	-	-	1.2	-	-	-	-
34	1606	dimethoxy-hydroxy-methyl-benzoquinone isomer 1	-	-	-	-	-	-	-	13.6	-	-	-	-
35	1653	dimethoxy-methylhydroquinone isomer 2	-	-	-	-	-	-	-	-	-	-	-	0.3
36	1680	dimethoxy-hydroxy-methyl-benzoquinone isomer 2	-	-	-	-	-	-	-	1.8	-	-	-	-
37	1691	dimethoxy-hydroxy-methyl-benzoquinone isomer 3	-	-	-	-	-	-	-	0,7	-	-	-	-

^a^Tb (*Typhloiulus bureschi*), Tg (*T. georgievi*), Tal (*T. aff. lobifer*), Tl (*T. lobifer*), Tn (*T. nevoi*), To (*T. orpheus*), Ts (*T. serborum*), Tnsp (*Typhloiulini* sp. n.), Sd (*Serboiulus deelemani*), Sk (*S. kresnik*), Sl (*S. lucifugus*), Ls (*Lamellotyphlus sotirovi*)

^b^The profile of *Typhloiulini sp. n*. was calculated as the mean of single profiles from 10 specimens as already published in Bodner et al. (2016)

Secretions of *T. bureschi*, *T. georgievi*, *T. orpheus*, and *Typhloiulini* sp. n. (Table 2) were analysed in the laboratory of the Zoological Institute, Graz, Austria, using a Trace GC coupled to a DSQ I mass spectrometer (Thermo Instruments, Vienna, Austria). The GC was equipped with a ZB-5 fused silica capillary column (30 m × 0.25 mm i.d., 0.25 μm film thickness, Phenomenex, Aschaffenburg, Germany). Injection was splitless with helium as carrier gas at 1.2 ml min^−1^. The temperature of the GC oven was held at 50 °C for 1 min and then programmed to 300 °C at 10 °C min^−1^, then held for 5 min at 300 °C. The ion source of the MS and the transfer line were kept at 200 °C and 310 °C, respectively. Electron impact (EI) spectra were recorded at 70 eV.

Gas chromatographic retention indices (RI) of compounds were calculated according to Van den Dool and Kratz (1963), using a standard mixture of *n*-alkanes (C_9_-C_36_) (SigmaAldrich, (Vienna, Austria). As a natural reference source for authentic 2-ethyl-1,4-benzoquinone, we used extracts of *Tribolium confusum* (Suzuki et al. 1988).

### Genetic Analyses

After extraction of defensive secretions, individuals were transferred into 99% ethanol and subsequently used for the genetic analyses. Two to five segments from the middle part of the bodies were dissected for DNA extraction by DNeasy Blood & Tissue Kit (Qiagen, Hilden, Germany). Primers used for the mitochondrial 16S rRNA gene were LR-J-12961 (Cognato and Vogler 2001) and LR-N-13398 (Simon et al. 1994) (Biomers, Ulm, Germany), and for the nuclear 28S rRNA gene, SH-28 and SL-28 (Muraji and Tachikawa 2000). We obtained 28S rDNA sequences for all species except for *T. serborum*. PCR amplifications were performed with annealing temperatures ranging from 45 °C – 55 °C using BioTherm™ Taq DNA Polymerase and 1.5 mM MgCl_2_ buffer. PCR purification was done with ExoSAP-IT (VWR, Langenfeld, Germany). For *T. bureschi* the PCR amplification was done with Phusiontaq and 7.5 mM MgCl_2_ buffer. PCR products were sequenced with 3.2 μmol amplification primers using the BigDye Terminator v.3.1 Cycle Sequencing Kit (Applied Biosystems, CA, USA) followed by purification of the product with Sephadex (VWR). Sequencing was performed in both directions on an automated capillary sequencer (ABI PRISM 3130xl; Applied Biosystems).

### Data Analysis

We deposited our sequences in GenBank (accession numbers can be found in supplementary Table 1) and downloaded available sequences for additional Julidae from GenBank (http://www.ncbi.nlm.nih.gov). Sequence alignment was performed by MUSCLE (Edgar 2004) in MEGA6 (Tamura et al. 2013). A total-evidence-tree was calculated. Sequences were combined into one alignment with a total length of 1073 bp. The 28S locus had a mean sequence length of 509 bp (longest and shortest sequences were 467 bp and 525 bp long, respectively). 16S sequences had a mean length of 427 bp (longest and shortest sequences were 394 bp and 449 bp long, respectively). The aligned 28S sequences had a total length of 559 bp: of those 559 bp 123 were constant, 45 were parsimony uninformative and 346 were parsimony informative. The aligned 16S sequences had a total length of 514 bp: of those 514 bp 425 were constant, 46 were parsimony uninformative and 88 were parsimony informative.

Phylogenetic inference was based on maximum likelihood (ML, 1500 ML-repetitions, 1000 BS-repetitions) and Bayesian inference (BI, 10,000,000 generations, 25% relative burnin). Analyses were performed by RAxML version 8.2 and by MrBayes version 3.2.6, respectively. PartitionFinder (Lanfear et al. 2012, 2014) selected the GTR + I + G model (for 16S) and the SYM + I + G model (for 28S) for BI and the GTR + I + G model (for 16S and 28S) for ML-analyses. Editing of phylogenetic trees was performed in FigTree version 1.4.2 (http://tree.bio.ed.ac.uk/software/figtree/). The phylogenetic trees herein shown are an extension of the current state of julid molecular systematics as published by Enghoff et al. (2011, 2013). Ancestral character state reconstruction was conducted in Mesquite Version 3.04 (Maddison and Maddison 2015), mapping distinct characters under an unordered maximum parsimony regime of equal-weighted gains and losses.

## Results

### Compound Identification

From all extracts, a total of 37 benzoquinones and related compounds were identified, all of which are considered to be part of the defensive secretions of the species investigated. A compound list for all species is provided in Table 1, and analytical data for all compounds are summarized in Supplementary Table 2. Most of the compounds were already familiar from previous studies, and their identification was carried out by a comparison to already known data sets (e.g. Bodner et al. 2016). These compounds included 1,4-benzoquinone (peak 1), a series of methyl- and/or methoxy-benzoquinones with or without hydroxyl group (peaks 3, 6, 8, 11, 13, 15, 16, 20, 21, 27, 28, 29, 30, 34, 36, 37), a series of methyl- and/or methoxy-substituted hydroquinones (peaks 14, 18, 24, 26, 32, 35), along with phenol (peak 2) and phenol derivatives (peaks 4, 19, 25).

Moreover, a subclass of benzohydroquinones and phenolics bearing an ethyl-group as alkyl-substituent was detected (peaks 5, 7, 10, 23,). The major component of this new subclass was compound 5, showing a molecular ion at m/z 136, along with fragments at m/z 108 (base ion), 107, 82, 80, 79 and 54, indicating a C_2_H_5_–1,4-benzoquinone. A fragment at m/z 54 is only consistent with a benzoquinone bearing substituent(s) on one side of the ring, thus limiting structure possibilities to 2,3-dimethyl-1,4-benzoquinone and 2-ethyl-1,4-benzoquinone, respectively. The mass spectrum of the compound completely matched that of 2-ethyl-1,4-benzoquinone from the NIST-library and from literature (e.g., Gnaspini and Cavalheiro 1998). Comparison of the compound’s retention index (measured RI = 1108) to indices reported from literature gave good correspondence to that of authentic 2-ethyl-1,4-benzoquinone (RI = 1103 in Rocha et al. 2013), and a clear difference to that of 2,3-dimethyl-1,4-benzoquinone (reported RI = 1119 in Rocha et al. 2013). As a natural reference for 2-ethyl-1,4-benzoquinone, we extracted the defensive secretion of *Tribolium confusum* (Coleoptera: Tenebrionidae), which has been reported to contain 2-ethyl-1,4-benzoquinone as a major compound (Suzuki et al. 1988), and showed this had identical mass spectrum and GC retention index to our sample compound.

Consequently, peaks 7 and 23 were identified as 4-ethyl-phenol (measured RI = 1177) and 2-ethyl-hydroquinone (measured RI = 1436), respectively, mainly on the basis of mass spectral data in combination with RIs from literature (e.g., El-Sayed et al. 2005). The RI reported for ethyl-hydroquinone, however, showed slight deviations (e.g., Rocha et al. 2013: RI = 1409).

Mass spectral data of peak no. 10 tentatively indicated 2-ethyl-3-methoxy-1,4-benzoquinone (measured RI = 1243), which was also supported by the co-occurrence of a similar compound, 2-methoxy-3-methyl-1,4-benzoquinone (peak 8).

Six compounds, all of which were trace or minor components (peaks 9 and 12, 17, 22, 31, 33), remained unidentified. Our chemical analyses also confirmed the presence of non-quinonic compounds, but their source and identification will be the subject of a future study.

### Secretion Profiles

All 12 species exhibited highly specific secretion profiles, as summarized in Table 2. In four species of mainly troglobiont *Typhloiulus* (*T. serborum, T. nevoi, T. bureschi, T. georgievi*) and both species of troglobiont *Serboiulus,* an abundant chemical fraction of ethyl-benzoquinones and related compounds was observed, with 2-ethyl-1,4-benzoquinone comprising up to 2/3 of individual secretions. Interestingly, *T. lobifer*, *T.* aff. *lobifer* and *Lamellotyphlus sotirovi*, the three of which are cave-dwellers too, completely lacked ethyl-benzoquinones. On the other hand, the secretions of *T. orpheus*, a representative of endogean typhloiulines, contained moderate amounts of ethyl-benzoquinones whereas no sign of these compounds was found in another (hitherto undescribed) endogean typhloiuline (“Typhloiulini” sp. n.). Ethyl-benzoquinones were not found in putatively close typhloiuline outgroups, such as in e.g. *Leptoiulus.*

In the 2-EB producing typhloiulines, EB-amounts ranged from 23% of the whole secretion (e.g., in *T. nevoi*) up to 66% of the secretion (e.g., in *S. lucifugus*), followed by 2-methyl-1,4-benzoquinone (from trace amounts to 35% of the secretion) and 2-methoxy-3-methyl-1,4-benzoquinone (from 1.4 to 74.1% of the secretion).

In the 2-EB lacking typhloiulines such as in the *T. lobifer*-group and in *Lamellotyphlus*, the common julid methoxy- and methyl-quinones predominated in the secretions (Table 2). Moreover, the secretion profiles of *T. lobifer, T. serborum* and *T. nevoi*, differed from all other species investigated in containing large amounts of 2-methyl-3,4-methylenedioxyphenol (13, 15 and 18%, respectively) and 2,3-dimethoxyhydroquinone (up to 10%).

### Phylogeny of “Typhloiulini” and the Evolutionary History of Ethyl-benzoquinones

**Fig. 2:**
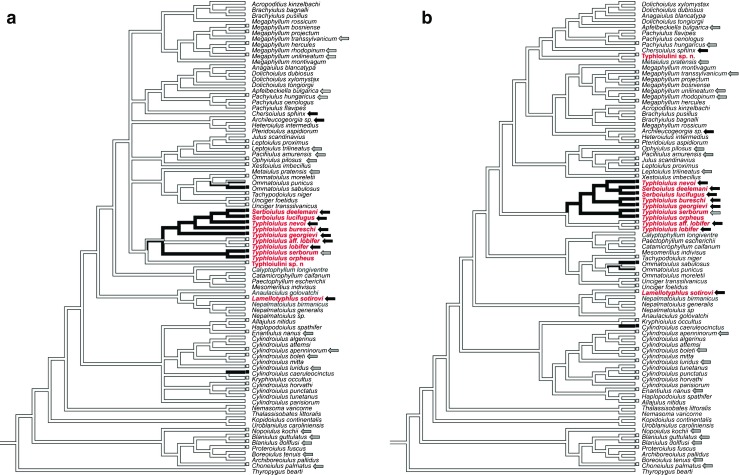
Ancestral character state reconstruction of character “ethyl benzoquinones” across the Julidae using the BI-tree (**a**) and the ML-tree (**b**). “Typhloiulini” investigated here in red. Cave-dwelling is indicated by black arrows; species that can be found in epi−/endogean habitats as well as in caves are indicated by grey arrows

In order to explain the taxonomic pattern of ethyl-benzoquinone occurrence, and to unravel whether a cave-effect triggered ethyl-benzoquinone evolution, we first classed the “Typhloiulini” investigated here within a phylogenetic framework, and then traced the evolutionary history of the character “ethyl-benzoquinones” in relation to cave-dwelling (Fig. 2).

In our phylogenetic (BI- and ML-) trees (Supplementary Figs. 1 and 2), the deeper level node support was weak due to the properties of the gene fragments analyzed. However, in both trees, the “Typhloiulini” sensu lato (i.e., typhloiulines in their traditional sense) were found to be polyphyletic, with *Typhloiulus* and *Serboiulus* being the sister group to a clade containing various species of 5 julid tribes, Brachyiulini, Pachyiulini, Leucogeorgiini, Julini, and (the remaining) Leptoiulini. Genus *Typhloiulus* was paraphyletic with respect to *Serboiulus*. The new (undescribed) typhloiuline species (“Typhloiulini sp. n.”) was found to be placed outside the clade containing *Typhloiulus* plus *Serboiulus*, either basal to these (ML: Supplementary Fig. 1) or as sister to *Metaiulus* (BI: Supplementary Fig. 2). *Lamellotyphlus* was placed far apart from the remaining typhloiulines in a basal julid clade also containing *Anaulaciulus* and *Nepalmatoiulus*. Within the clade containing *Typhloiulus* plus *Serboiulus*, the *T. lobifer*-species group splits off early, thus being the sister to a lineage containing the remaining *Typhloiulus-* and *Serboiulus*-species (ML). In BI, the same group shows a trichotomy: *(i) Serboiulus* spp. plus *Typhloiulus nevoi*, *T. bureschi*, *T. gerogievi*; *ii) T. lobifer*-group; *iii) T. serborum* plus *T. orpheus*). However, basically, ML- and BI-hypotheses of julid phylogeny supported a very similar phylogenetic structure. In both trees, the position of *Lamellotyphlus* and “Typhloiulini*”* sp. n. clearly implies polyphyly of typhloiulines in their traditional sense (“Typhloiulini” sensu lato).

Trogobiism/trogloxenism is scattered all across the Julida (Fig. 2, black arrows) and obviously evolved multiple times independently. In “Typhloiulini” sensu lato, cave dwelling is found in *Serboiulus* plus a part of *Typhloiulus* and in *Lamellotyphlus*. For the clade containing *Typhloiulus* and *Serboiulus*, a general tendency to troglobiism is indicated, even though not all species (e.g., *T. orpheus;* partly *T. serborum*) are troglobionts.

Ethyl-benzoquinones (EBs) characterized both cave-dwelling *(T. nevoi, T. bureschi, T. georgievi, T. serborum, Serboiulus* spp.) and non-cave-dwelling species (e.g., *T. orpheus*). Mapping the character “ethyl-benzoquinones” onto our phylogenies suggested a single introduction of EBs in “typhloiulines” (Fig. 2). In the ML-tree, EBs even characterized a distinct clade comprising *Typhloiulus nevoi, T. bureschi, T. georgievi, T. serborum, T. orpheus* and all species of *Serboiulus,* but not *T. lobifer, T. aff. lobifer* nor the new *“*Typhloiulini*”* sp. n. (Fig. 2B). It is also noteworthy that the EB-producing Typhloiulini include all analyzed species of subgenus *Typhloiulus* sensu stricto, as defined by Strasser (1962, 1966) and complemented by Vagalinski et al. (2015), but exclude the two remaining species of *Typhloiulus* of unclear subgeneric affiliation – *T. lobifer* and *T. aff. lobifer*. In the BI-tree, a compact EB-clade is blurred by the trichotomy of this group of typhloiulines (as mentioned above), and EBs characterize two (of the three) subgroups (Fig. 2a).

## Discussion

### Ethyl-benzoquinones: a Novel Subclass of Benzoquinones in the Julida

To date, repugnatorial secretions have been analyzed for more than 40 representatives of Julida, including species from epigeic, endogeic, and arboricolous habitats (e.g., Bodner and Raspotnig 2012; Bodner et al. 2016; Huth 2000; Sekulić et al. 2014; Shear 2015; Vujisić et al. 2011). The secretions of all these species have been shown to rely mainly on methyl- and/or methoxy-benzoquinones, with only a few species producing additional, very specific non-benzoquinone compounds (e.g., Bodner and Raspotnig 2012; Huth 2000; Shimizu et al. 2012;). The two most common defensive components appear to be 2-methyl-1,4-benzoquinone (toluquinone) and 2-methoxy-3-methyl-1,4-benzoquinone (MMBQ). These compounds typically prevail in julid secretions, and the latter compound has even been detected in all species hitherto analyzed (with the exception of an early, probably incomplete analysis of *Julus terrestris* by Béhal and Phisalix 1900). Such a homogenous chemistry in epi- and endogean julid millipedes provides a good basis for a comparison to the secretions of cavernicolous species.

“Typhloiulines” – at least some of them – are obviously different: the most abundant compound in the *Typhloiulus* sensu stricto- plus *Serboiulus*-group was 2-ethyl-1,4-benzoquinone (2-EB), a new main compound for the secretions of julidans. Trace amounts of 2-EB have sporadically been reported from a few juliformians, but these rather represent by-products of major methyl- and/or methoxy-substituted benzoquinones. This is not comparable to the situation in “Typhloiulini”. Non-typhloiuline species showing traces of 2-EB include the spirostreptid *Telodeinopus aoutii* (Deml and Huth 2000), the spirobolidans *Acladocricus setigerus* and *Rhinocricus varians* (Moussatche et al. 1969; Wu et al. 2007), as well as the julids *Cylindroiulus caeruleocinctus*, *Unciger transsilvanicus* and *Ommatoiulus sabulosus* (Huth 2000; Röper and Heyns 1977; Sekulić et al. 2014). Interestingly, exclusively in members of subgenus *Typhloiulus* sensu stricto plus *Serboiulus* – thus in a distinct part of the “Typhloiulini” sensu lato only – EBs represent the leading benzoquinone compounds. This correlates with the results from the phylogenetic analyses where subgenus *Typhloiulus* sensu stricto plus *Serboiulus* forms a compact clade (Supplementary Figs. 1 and 2). We thus consider that the evolution of EBs and related compounds represents a condition derived from the common methyl-benzoquinones in the Julida. Outside the Diplopoda, ethyl-benzoquinones are not rare, and well-known from a diversity of beetles, mainly tenebrionids (e.g., Suzuki et al. 1988), dermapterans (Schildknecht and Weiss 1960), and certain cave- and non-cave-dwelling laniatorean Opiliones (e.g., Gnaspini and Cavalheiro 1998; Rocha et al. 2013).

### “Typhloiulini” and Troglobiism

“Typhloiulini” in the traditional sense are not a monophyletic group, as evidenced by our phylogenetic analyses. Traditional “Typhloiulini” (we here referred to these as Typhloiulini sensu lato) currently comprises eight genera with 57 species distributed from French Maritime Alps in the west to Bulgaria and Romania in the east, and from Austria in the north to Sicily and the Peloponnesus in the south (Mauries et al. 1997; Strasser 1962; Tabacaru et al. 2002; Vagalinski et al. 2015). Species belonging to the genera *Trogloiulus* (Manfredi 1931), *Serboiulus* (Strasser 1962), *Alpityphlus* (Strasser 1967), *Banatoiulus* (Tabacaru 1985), and *Lamellotyphlus* (Tabacaru 1976) are exclusively cave-dwellers. Representatives of the genera *Typhloiulus* (Latzel 1884), *Leptotyphloiulus* (Verhoeff 1899), and *Buchneria* (Verhoeff 1941) include both cavernicolous and geophile (or petrophilous) forms (Mauries et al. 1997; Strasser 1962). Some of the species e. g. *Typhloiulus tobias* (Berlese 1886), *T. maximus* (Verhoeff 1929), *T. albanicus* (Attems 1929), *T. kotelensis* (Jawłowski 1938), have been found in caves, but also in epigean habitats. There are different opinions concerning the position of the tribe “Typhloiulini”. Strasser (1962) revised whole group of “Typhloiulini” and explained that they deserve tribal level. Mauries et al. (1997) described a new typhloiuline from Albania and assumed that "… there are no apomorphies whatever in typhloiulines which would distinguish them from … tribe Leptoiulini". However, Tabacaru et al. (2002) did not agree with such synonymy and retained the tribus Typhloiulini, as well as Shelley et al. (2000). If we exclude genus *Leptotyphloiulus*, all other typhloiulines share the absence of a coxal piece and the absence of phylacum, respectively. Furthermore, in numerous species, mostly belonging to *Typhloiulus*, a specifically fringed lamella between the mesomerite and opisthomerite is present (Tabacaru et al. 2002). In any case, typhloiulines are frequently found in hypogean habitats, and tend to exhibit troglomorphism, such as reduction of eyes, elongation of appendages or reduction of pigmentation. Several typhloiuline species are true troglobionts, probably using caves as permanent habitats and also as reproductive sites; this is indicated by the finding of early postembryonic stages in different cave systems (e. g. *Serboiulus lucifugus* Strasser 1962; *S. deelemani* Strasser, 1972; *Lamellotyphlus belavodae* Makarov et al. 2008).

### EB-production in Typhloiulines and the Absence of a *Cave*-effect

In outgroups to the EB-producing typhloiuline lineage, such as in the *T. lobifer*-group (cavernicolous), “Typhloiulini” sp. n. (epi−/endogean) or in several leptoiulines (epigean), no trace of EBs has been detected (see also Vujisić et al. 2011). By contrast, also non cave-dwellers such *T. orpheus* and species found in- and outside caves (such as *T. serborum*) produce EBs. Thus, there is no convincing correlation between cave-dwelling and EB-production (Fig. 2). This argument is substantiated by the chemistry of many non-typhloiuline cave-dwellers: none of them produces EBs (Fig. 2). On the other hand, EBs as minor or trace components have also been acquired by some non-typhloiulines such as *Cylindroiulus caeruleocintus* and *Ommatoiulus sabulosus*. These are, however, non-cave dwellers.

Transitions to cave-dwelling certainly represent a derived trait in millipedes, and cave-dwelling is shown to have evolved several times independently in the Julida (Fig. 2). Of the 82 species shown in our trees, 28 are, at least potentially, found in caves. For the “Typhloiulini” sensu lato, we consider troglobiism as being independently acquired many times even though a tendency to cave-dwelling may characterize clades such as *Typhloiulus* plus *Serboiulus*, with some exceptions. We, however, avoided mapping “troglobiism” onto our trees, because “troglobiism” is no character *per se* and has to be interpreted with caution: troglobiont species frequently represent endemites of distinct caves, indicating that each species independently found entrance into its particular cave. In typhloiulines, e.g. in *Typhloiulus* plus *Serboiulus*, a general tendency to borrow into deep habitats may have evolved early, rather than troglobiism itself. Adaptations to such deep endogean habitats such as the MSS (mesovoid shallow stratum) may indeed have preceded cave-dwelling in many other diplopods as well (Liu et al. 2017).

Biogeographically, all analyzed species of this EB-lineage belong to the Carpatho-balkanic and Rhodopean fauna. On the other hand, the *T. lobifer-*group (without a trace of ethyl-benzoquinones), belongs to the Dinaric faunal elements. Such a difference in secretion profiles in biogeographically separated groups of typhloiulines supports EBs as a phylogenetic signal. In this respect, our data indicate that biogeographically-separated lineages might have undergone a distinctly different evolution regarding their chemistry. Possibly, only the Carpatho-balkanic/Rhodopean lineage developed EBs.

In the opposite scenario and if EB-development was driven by a cave-environment, EBs would result from parallelism, and EB-evolution/−regression would have occurred several times convergently in closely-related species. Referring to evolutionary parsimony, such a scenario is unlikely. It is more likely that EBs already evolved early in a common ancestor of the Carpatho-balkanic/Rhodopian lineage. Subsequently, this lineage diversified into many species, some of which independently found entrance into caves.

Comparably, in cave-dwelling representatives of other arthropod groups, the particular environment of caves did not seem to have much affect the composition of secretions. A study on the Texas cave harvestmen *Chinquipellobunus madlae* (Shear et al. 2010a), for instance, revealed a secretion chemically well-fitting the chemosystematics of this group, not showing a “cave-effect”. The same was found for the troglobiont callipodidan diplopod *Tetracion jonesi* (Abacionidae), producing phenolic compounds for defense just like its epigean relatives (Shear et al. 2010b). Comparably, Makarov et al. (2010, 2012) reported on a widely homogenous cyanogenic chemistry in both cave-dwelling and epigean Polydesmida. Thus, based on currently available data, and also supported by our study, the chemistry of defensive secretions is not easily affected by troglobiism. Furthermore, all these examples may support a general and remarkable chemical conservatism of defensive secretions, as already shown for a diversity of taxa (e.g., Raspotnig et al. 2012).

## Electronic supplementary material

Supplementary Fig 1 (PDF 1360 kb)

Supplementary Fig 2 (PDF 1920 kb)

Supplementary Table 1 (DOC 142 kb)

Supplementary Table 2 (DOC 88 kb)
