# Supplementary figures and images for: Chemical Ecology of Cave-Dwelling Millipedes: Defensive Secretions of the Typhloiulini (Diplopoda, Julida, Julidae)
Source: J Chem Ecol. 2017 Mar 16;43(4):317–26. doi: 10.1007/s10886-017-0832-1 (PMC5399059; doi:10.1007/s10886-017-0832-1)

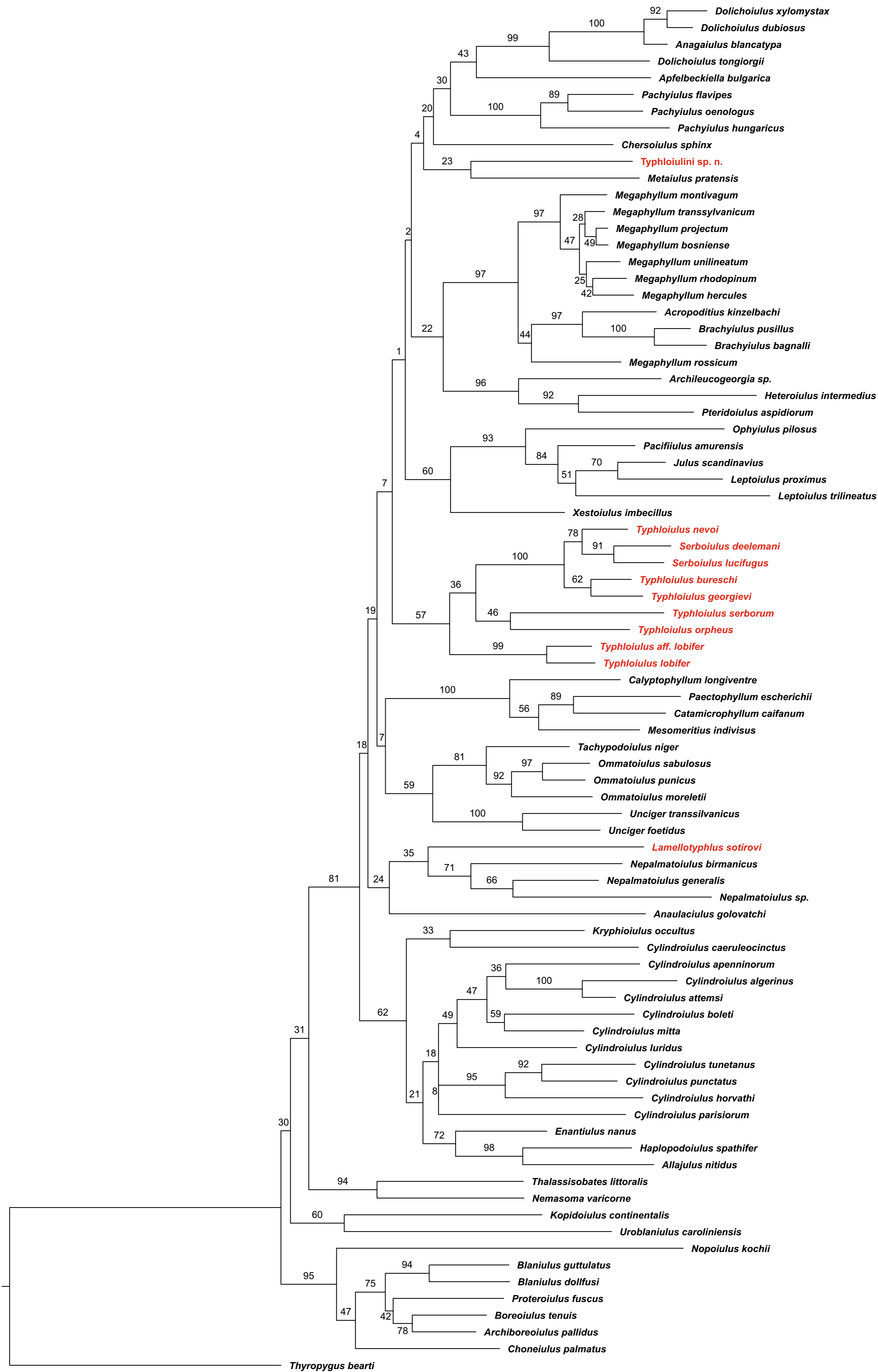

0.2

Supplement: Supplementary file 1 — (PDF 1360 kb) [file 10886_2017_832_MOESM1_ESM.pdf]

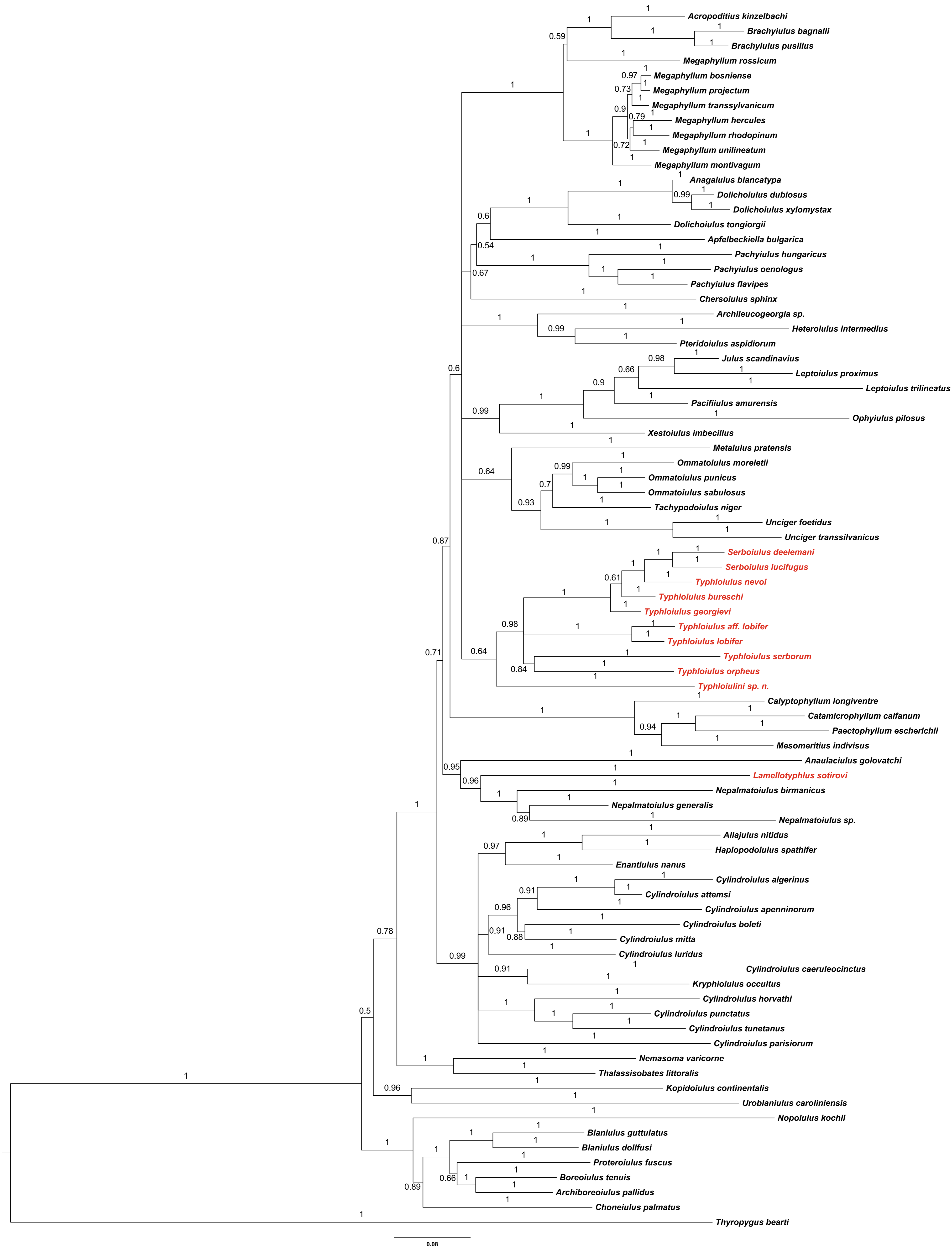

Supplement: Supplementary file 2 — (PDF 1920 kb) [file 10886_2017_832_MOESM2_ESM.pdf]
