# Supplementary material for: Chemical Ecology of Cave-Dwelling Millipedes: Defensive Secretions of the Typhloiulini (Diplopoda, Julida, Julidae)
Source: J Chem Ecol. 2017 Mar 16;43(4):317–26. doi: 10.1007/s10886-017-0832-1 (PMC5399059; doi:10.1007/s10886-017-0832-1)
Supplement: Supplementary file 3 — (DOC 142 kb) [file 10886_2017_832_MOESM3_ESM.doc]

Supplementary Table 1. Species list for phylogenetic analyses, voucher numbers and GenBank accession codes

| **Order** | **Family** | **Tribe** | **Species** | **Accession Number NCBI: 28s** | **Accession Number NCBI: 16s** | **Voucher Number** |
| --- | --- | --- | --- | --- | --- | --- |
| Julida | Blaniulidae | Blaniulini | *Blaniulus dollfusi* | JF321043 | JF320988 | ZMUC00100954 |
|  |  |  | *Blaniulus guttulatus* | JF321015 | JF320960 | ZMUC00200020 |
|  |  | Boreoiulini | *Archiboreoiulus pallidus* | KF701088 | KF701063 | ZMUC00101178 |
|  |  |  | *Boreoiulus tenuis* | JF321060 | JF321005 | ZMUC00101005 |
|  |  |  | *Proteroiulus fuscus* | JF321028 | JF320973 | ZMUC00101185 |
|  |  | Choneiulini | *Choneiulus palmatus* | JF321044 | JF320989 | ZMUC00100758 |
|  |  | Nopoiulini | *Nopoiulus kochii* | KF701108 | KF701083 | ZMUC00103127 |
|  | Julidae | Brachyiulini | *Acropoditius kinzelbachi* | KF701110 | KF701085 | ZMUC00021405 |
|  |  |  | *Anaulaciulus golovatchi* | JF321036 | JF320981 | ZMUC00100963 |
|  |  |  | *Brachyiulus bagnalli* | JF321039 | JF320984 | ZMUC00100790 |
|  |  |  | *Brachyiulus pusillus* | JF321038 | JF320983 | ZMUC00100789 |
|  |  |  | *Megaphyllum bosniense* | KF701098 | KF701073 | ZMUC00021391 |
|  |  |  | *Megaphyllum hercules* | KF701100 | KF701075 | ZMUC00021393 |
|  |  |  | *Megaphyllum montivagum* | KF701092 | KF701067 | ZMUC00101492 |
|  |  |  | *Megaphyllum projectum* | JF321018 | JF320963 | ZMUC00200023 |
|  |  |  | *Megaphyllum rhodopinum* | KF701101 | KF701076 | ZMUC00021394 |
|  |  |  | *Megaphyllum rossicum* | KF701094 | KF701069 | ZMUC00101119 |
|  |  |  | *Megaphyllum transsylvanicum* | KF701099 | KF701074 | ZMUC00021392 |
|  |  |  | *Megaphyllum unilineatum* | KF701104 | KF701079 | ZMUC00021397 |
|  |  | Calyptophyllini | *Calyptophyllum longiventre* | JF321021 | JF320966 | ZMUC00200148 |
|  |  | Cylindroiulini | *Allajulus nitidus* | JF321023 | JF320968 | ZMUC00200175 |
|  |  |  | *Cylindroiulus algerinus* | JF321047 | JF320992 | ZMUC00101061 |
|  |  |  | *Cylindroiulus apenninorum* | JF321049 | JF320994 | ZMUC00101014 |
|  |  |  | *Cylindroiulus attemsi* | JF321048 | JF320993 | ZMUC00101058 |
|  |  |  | *Cylindroiulus boleti* | JF321017 | JF320962 | ZMUC00200022 |
|  |  |  | *Cylindroiulus caeruleocinctus* | JF321009 | JF320954 | ZMUC00101181 |
|  |  |  | *Cylindroiulus horvathi* | JF321040 | JF320985 | ZMUC00100791 |
|  |  |  | *Cylindroiulus luridus* | JF321042 | JF320987 | ZMUC00100793 |
|  |  |  | *Cylindroiulus mitta* | JF321050 | JF320995 | ZMUC00101064 |
|  |  |  | *Cylindroiulus parisiorum* | JF321051 | JF320996 | ZMUC00101031 |
|  |  |  | *Cylindroiulus punctatus* | JF321014 | JF320959 | ZMUC00200019 |
|  |  |  | *Cylindroiulus tunetanus* | JF321052 | JF320997 | ZMUC00101065 |
|  |  |  | *Enantiulus nanus* | JF321032 | JF320977 | ZMUC00100754 |
|  |  |  | *Kryphioiulus occultus* | JF321034 | JF320979 | ZMUC00100757 |
|  |  | Julini | *Haplopodoiulus spathifer* | JF321058 | JF321003 | ZMUC00101004 |
|  |  |  | *Julus scandinavius* | JF321012 | JF320957 | ZMUC00200018 |
|  |  |  | *Pacifiiulus amurensis* | JF321035 | JF320980 | ZMUC00100959 |
|  |  | Leptoiulini | *Leptoiulus proximus* | JF321016 | JF320961 | ZMUC00200021 |
|  |  |  | *Leptoiulus trilineatus* | JF321041 | JF320986 | ZMUC00100792 |
|  |  |  | *Ophyiulus pilosus* | JF321024 | JF320969 | ZMUC00200176 |
|  |  |  | *Xestoiulus imbecillus* | KF701093 | KF701068 | ZMUC00101120 |
|  |  | Leucogeorgiini | *Archileucogeorgia sp* | KF701091 | KF701066 | ZMUC00101662 |
|  |  |  | *Heteroiulus intermedius* | JF321019 | JF320964 | ZMUC00200077 |
|  |  |  | *Nepalmatoiulus birmanicus* | KF701109 | KF701084 | ZMUC00101354 |
|  |  |  | *Nepalmatoiulus generalis* | KF701090 | KF701065 | ZMUC00101661 |
|  |  |  | *Nepalmatoiulus sp.* | JF321020 | JF320965 | ZMUC00200145 |
|  |  | Metaiulini | *Metaiulus pratensis* | KF701089 | KF701064 | ZMUC00101657 |
|  |  | Oncoiulini | *Unciger foetidus* | JF321033 | JF320978 | ZMUC00100755 |
|  |  |  | *Unciger transsilvanicus* | KF701102 | KF701077 | ZMUC00021395 |
|  |  | Pachyiulini | *Anagaiulus blancatypa* | JF321056 | JF321001 | ZMUC00101029 |
|  |  |  | *Apfelbeckiella bulgarica* | KF701105 | KF701080 | ZMUC00021398 |
|  |  |  | *Chersoiulus sphinx* | KF701095 | KF701070 | ZMUC00101686 |
|  |  |  | *Dolichoiulus dubiosus* | JF321062 | JF321007 | ZMUC00200138 |
|  |  |  | *Dolichoiulus tongiorgii* | JF321026 | JF320971 | ZMUC00200178 |
|  |  |  | *Dolichoiulus xylomystax* | JF321061 | JF321006 | ZMU00101028C |
|  |  |  | *Pachyiulus hungaricus* | KF701103 | KF701078 | ZMUC00021396 |
|  |  |  | *Pachyiulus varius* | JF321037 | JF320982 | ZMUC00101187 |
|  |  |  | *Pachyiulus varius* | JF321055 | JF321000 | ZMUC00101011 |
|  |  | Paectophyllini | *Catamicrophyllum caifanum* | JF321057 | JF321002 | ZMUC00101037 |
|  |  |  | *Mesomeritius indivisus* | KF701107 | KF701082 | ZMUC00021387 |
|  |  |  | *Paectophyllum escherichii* | KF701096 | KF701071 | ZMUC00101712 |
|  |  | Pteridoiulini | *Pteridoiulus aspidiorum* | KF701112 | KF701087 | ZMUC00021417 |
|  |  | Schizophyllini | *Ommatoiulus moreletii* | JF321013 | JF320958 | ZMUC00200101 |
|  |  |  | *Ommatoiulus punicus* | JF321053 | JF320998 | ZMUC00101056 |
|  |  |  | *Ommatoiulus sabulosus* | JF321010 | JF320955 | ZMUC00101182 |
|  |  |  | *Tachypodoiulus niger* | JF321025 | JF320970 | ZMUC00200177 |
|  |  | Typhloiulini | ***Lamellotyphlus sotirovi*** | KY655322 | KY655314 | FBIZO1120* |
|  |  |  | ***Serboiulus deelemani*** | KY701736 | KY701734 | FBIZO1160* |
|  |  |  | ***Serboiulus lucifugus*** | KY701737 | KY701735 | FBIZO1180* |
|  |  |  | ***Typhloiulus nevoi*** | KY655327 | KY655319 | FBIZO1140* |
|  |  |  | ***Typhloiulus bureschi*** | KY655323 | KY655315 | NHMW 8683 (1♀)* |
|  |  |  | ***Typhloiulus aff. lobifer*** | KY655324 | KY655316 | FBIZO10017* |
|  |  |  | ***Typhloiulus georgievi*** | KY655325 | KY655317 | NHMW 8684 (1♂)* |
|  |  |  | ***Typhloiulus lobifer*** | KY655326 | KY655318 | FBIZO1130* |
|  |  |  | ***Typhloiulus serborum*** | --- | KY655320 | FBIZO1150* |
|  |  |  | *Typhloiulus orpheus* | KF701106 | KF701081 | ZMUC00021400 |
|  |  |  | ***Typhloiulini sp. n.*** | KY655328 | KY655321 | in process of description (Vagalinski et al., unpublished) |
|  | Parajulidae |  | *Uroblaniulus caroliniensis* | JF321059 | JF321004 | ZMUC00101026 |
|  | Mongoliulidae |  | *Kopidoiulus continentalis* | JF321046 | JF320991 | ZMUC00100957 |
|  | Nemasomatidae |  | *Nemasoma varicorne* | JF321030 | JF320975 | ZMUC00101183 |
|  |  |  | *Thalassisobates littoralis* | KF701097 | KF701072 | ZMUC00101123 |
| Spirostreptida | Harpagophoridae |  | *Thyropygus bearti* | JF321008 | JF320953 | ZMUC00200141 |

Only species in **bold** were investigated in this study. Meta-data for the remaining taxa are from Enghoff et al. (2011, 2013). *Voucher deposition & museum/institution acronyms: NHMW (Natural History Museum of Vienna), NMNHS (National Museum of Natural History Sofia), FBIZO (Faculty of Biology, Institute of Zoology, University of Belgrade)
