# Supplementary material for: Chemical Ecology of Cave-Dwelling Millipedes: Defensive Secretions of the Typhloiulini (Diplopoda, Julida, Julidae)
Source: J Chem Ecol. 2017 Mar 16;43(4):317–26. doi: 10.1007/s10886-017-0832-1 (PMC5399059; doi:10.1007/s10886-017-0832-1)
Supplement: Supplementary file 4 — (DOC 88 kb) [file 10886_2017_832_MOESM4_ESM.doc]

Supplementary Table 2: Gas chromatographic and mass spectral data

| peak no. | retention index RI  measured authentic reference* | | mass spectrometric fragmentation  *m/z (relative intensity)* |  | identified as |
| --- | --- | --- | --- | --- | --- |
|  |  |  |  |  |  |
| 1 | 917 | 920 | 110 ([M+2]^+^, 5), 108 (M^+^,100), 82 (20), 80 (22), 54 (34) |  | 1,4-benzoquinone |
| 2 | 977 | 978 | 95 ([M+1]^+^, 5), 94 (M^+^,100), 66 (16), 65 (14), 57 (5), 55 (3) |  | phenol |
| 3 | 1015 | 1014 | 122 (M^+^, 100), 94 (54), 82 (28), 68 (16), 66 (22), 54 (31) |  | 2-methyl-1,4-benzoquinone |
| 4 | 1071 | 1071 | 108 (M^+^, 100), 107 (82), 90 (6), 80 (6), 79 (11), 77 (13) |  | *p*-cresol |
| 5 | 1108 | 1103^a^ | 136 (M^+^, 77), 108 (100), 107 (38), 82 (24), 79 (40), 65 (7), 54 (31) |  | 2-ethyl-1,4-benzoquinone |
| 6 | 1120 | 1120 | 138 (M^+^,100), 110 (11), 83 (15), 82 (40), 81 (20), 55 (11), 54 (10) |  | 2-hydroxy-3-methyl-1,4-benzoquinone |
| 7 | 1177 | 1178^b^ | 122 (M^+^, 36), 108 (9), 107 (100), 91 (6), 77 (16), 65 (5), 51 (4) |  | *p*-ethylphenol |
| 8 | 1182 | 1183 | 152 (M^+^, 100), 151 (20), 137 (5), 123 (7), 122 (47), 109 (13), 94 (5), 83 (8), 82 (12), 81 (7), 67 (8), 66 (15), 54 (10), 53 (12) |  | 2-methoxy-3-methyl-1,4-benzoquinone |
| 9 | 1191 | - | 152 (M^+^, 100), 137 (3), 123 (3), 109 (6), 96 (7), 95 (14), 83 (8), 82 (13), 81 (12), 78 (13), 69 (18), 55 (11) |  | unidentified |
| 10 | 1243 | - | 166 (M^+^, 100), 151 (70), 138 (10), 133 (17), 123 (62), 121 (13), 95 (18), 82 (9), 79 (11), 69 (10), 67 (11), 54 (10) |  | 2-ethyl-3-methoxy-1,4-benzoquinone |
| 11 | 1245 | - | 138 (M^+^, 61), 123 (14), 110 (64), 108 (62), 95 (37), 82 (38), 69 (100), 54 (35), 53 (28), 52 (23) |  | 2-methoxy-1,4-benzoquinone |
| 12 | 1317 | - | 150 (M^+^, 16), 122 (69), 94 (37), 82 (100), 68 (18), 66 (43), 65 (9), 54 (93), 53 (19), 40 (24) |  | unidentified |
| 13 | 1319 | 1320 | 168 (M^+^, 64), 153 (31), 125 (17), 123 (100), 97 (13), 95 (15), 82 (36), 69 (65), 54 (37), 53 (13) |  | 2,3-dimethoxy-1,4-benzoquinone |
| 14 | 1341 | 1341 | 124 (M^+^,100), 123 (44), 107 (13), 105 (6), 95 (29), 77 (15), 69 (4), 67 (13), 57 (16) |  | 2-methylhydroquinone |
| 15 | 1341 | - | 152 (M^+^,78), 137 (15), 124 (41), 123 (36), 122 (29), 109 (11), 84 (10), 69 (100), 66 (24), 56 (4) |  | 2-methoxy-5-methyl-1,4-benzoquinone |
| 16 | 1346 | - | 152 (M^+^, 70 ), 137 (14), 124 (87), 123 (18), 122 (68), 109 (22), 96 (16), 94 (12), 81 (8), 69 (100), 66 (28), 53 (26) |  | 2-methoxy-6-methyl-1,4-benzoquinone |
| 17 | 1349 | - | 151 (M^+^,62), 120 (20), 119 (100), 92 (28), 91 (4), 65 (4), 63 (5) |  | unidentified |
| 18 | 1375 | 1373^c^ | 170 (M^+^, 100), 155 (50), 123 (6), 112 (14), 109 (24), 95 (5), 81 (4), 57 (16) |  | 2,3-dimethoxyhydroquinone |
| 19 | 1386 | 1390^c^ | 152 (M^+^, 100), 151 (88), 136 (9), 121 (15), 66 (13), 65 (11) |  | 2-methyl-3,4-methylenedioxyphenole |
| 20 | 1411 | - | 154 (M^+^,100 ), 139 (27), 128 (13), 126 (50), 111 (15), 98 (5), 97 (5), 85 (1), 82 (75), 72 (15), 57 (7), 54 (51) |  | 2-hydroxy-3-methoxy-1,4-benzoquinone |
| 21 | 1421 | 1420 | 182 (M^+^, 84), 167 (45), 153 (22), 139 (25), 137 (100), 136 (20), 121 (10), 111 (20), 108 (6), 96 (11), 83 (39), 69 (17), 68 (21), 67 (9) |  | 2,3-dimethoxy-5-methyl-1,4-benzoquinone |
| 22 | 1422 | - | 165 (M^+^, 100), 134 (17), 133 (26), 132 (36), 116 (5), 106 (8), 105 (49), 104 (44), 91 (3), 78 (8), 77 (15), 66 (4) |  | unidentified |
| 23 | 1436 | 1409^a^ | 138 (M^+^, 57), 124 (8), 123 (100), 91 (5), 77 (6), 67 (9), 65 (6), 55 (5) |  | 2-ethyl-hydroquinone |
| 24 | 1449 | - | 184 (M^+^, 100), 182 (8), 169 (71), 154 (11), 141 (12), 137 (20), 126 (41), 123 (19), 69 (11), 83 (12), 69 (23) |  | dimethoxy-methylhydroquinone /isomer 1 |
| 25 | 1455 | 1456 | 152 (M^+^, 58), 122 (6), 121 (100), 93 (12), 92 (1), 65 (6) |  | methylparaben |
| 26 | 1465 | - | 166 (M^+^, 93), 165 (20), 152 (9), 151 (100), 121 (23), 77 (10), 65 (9), 53 (10) |  | 2,3,5,6-tetramethylhydroquinone |
| 27 | 1499 | - | 184 (M^+^,50), 169 (100), 141 (7), 139 (2), 127 (2), 123 (22), 113 (9), 87 (20), 85 (15), 69 (4), 68 (3), 54 (6) |  | dimethoxy-hydroxy-benzoquinone isomer |
| 27 | 1511 | - | 182 (M^+^, 100), 167 (13), 149 (14), 139 (79), 137 (8), 111 (32), 109 (24), 96 (11), 94 (15), 83 (90), 69 (79), 67 (21), 55 (14), 53 (22), 41 (10) |  | 2,6-dimetoxy-3-methyl-1,4-benzoquinone |
| 29 | 1518 | - | 168 (M^+^, 100), 153 (23), 140 (70), 125 (23), 112 (9), 97 (14), 96 (12), 85 (24), 72 (11), 68 (34) |  | 2-hydroxy-3-methoxy-5-methyl-1,4-benzoquinone |
| 30 | 1522 | - | 196 (M^+^, 97), 182 (22), 181 (59), 153 (31), 151 (100), 125 (33), 123 (18), 97 (57) 82 (26), 69 (24), 53 (31) |  | 2,3-dimethoxy-5,6-dimethyl-1,4-benzoquinone |
| 31 | 1532 | - | 214 (M^+^, 73), 169 (19), 168 (37), 167 (11), 155 (8), 138 (45), 137 (15), 136 (15), 127 (100), 114 (8), 99 (22), 68 (16), 59 (24) |  | unidentified |
| 32 | 1535 | - | 199 ([M+1]^+^, 10), 198 (M^+^, 90), 184 (9), 183 (100), 168 (24), 151 (5), 140 (5), 123 (9), 69 (5) |  | 2,3-dimethoxy-5,6-dimethylhydroquinone |
| 33 | 1598 | - | 200 (M^+^, 100), 185 (15), 151 (50), 139 (4), 123 (18), 85 (2), 69 (4) |  | unidentified |
| 34 | 1606 | - | 198 (M^+^, 100), 183 (83), 165 (27), 155 (16), 153 (10), 152 (5), 137 (32), 127 (20), 123 (4), 109 (7), 99 (27), 96 (7), 87 (22), 83 (5), 72 (3), 68 (10) |  | dimethoxy-hydroxy-methyl-benzoquinone isomer 1 |
| 35 | 1653 | - | 184 (M^+^, 100), 182 (28), 169 (43),141 (54), 139 (34), 126 (33), 111 (20), 83 (37), 69 (56), 67 (19), 53 (21) |  | dimethoxy-methylhydroquinone isomer 2 |
| 36 | 1680 | - | 198 (M^+^, 100), 183 (21), 180 (3), 170 (7), 165 (7), 155 (26), 152 (4), 140 (4), 137 (4), 127 (15), 125 (5), 112 (3), 109 (8), 99 (17), 85 (14), 83 (14), 72 (6) |  | dimethoxy-hydroxy-methyl-benzoquinone isomer 2 |
| 37 | 1691 | - | 198 (M^+^, 100), 183 (69), 180 (7), 170 (32), 169 (26), 165 (8), 155 (12), 151 (6), 127 (14), 125 (43), 112 (9), 109 (4), 99 (27), 85 (33), 83 (44), 82 (4), 72 (4), 67 (11) |  | dimethoxy-hydroxy-methyl-benzoquinone isomer 3 |
|  |  |  |  |  |  |

*Authentic standards (materials and methods) were used for index-calculation. In cases where no standards were available, we compared the RIs measured for particular secretion components to RIs reported in literature, as ^a^Rocha et al. (2013), ^b^El-Sayed et al. (2005), ^c^Vujisić et al. (2011).
